# Evaluating the Effect of Topical Atropine Use for Myopia Control on Intraocular Pressure by Using Machine Learning

**DOI:** 10.3390/jcm10010111

**Published:** 2020-12-30

**Authors:** Tzu-En Wu, Hsin-An Chen, Mao-Jhen Jhou, Yen-Ning Chen, Ting-Jen Chang, Chi-Jie Lu

**Affiliations:** 1 Department of Ophthalmology, Shin Kong Wu Ho-Su Memorial Hospital, Taipei 11101, Taiwan; wujessica168@yahoo.com; 2 School of Medicine, Fu Jen Catholic University, New Taipei City 242062, Taiwan; 3 School of Medicine, Chang Gung University, Taoyuan City 33302, Taiwan; chendavepic@gmail.com (H.-A.C.); annie850924@gmail.com (Y.-N.C.); 4 Graduate Institute of Business Administration, Fu Jen Catholic University, New Taipei City 242062, Taiwan; aaa73160@gmail.com (M.-J.J.); metheus.c@gmail.com (T.-J.C.); 5 Artificial Intelligence Development Center, Fu Jen Catholic University, New Taipei City 242062, Taiwan; 6 Department of Information Management, Fu Jen Catholic University, New Taipei City 242062, Taiwan

**Keywords:** intraocular pressure (IOP), topical atropine, myopia, machine learning, abbreviations and acronyms

## Abstract

Atropine is a common treatment used in children with myopia. However, it probably affects intraocular pressure (IOP) under some conditions. Our research aims to analyze clinical data by using machine learning models to evaluate the effect of 19 important factors on intraocular pressure (IOP) in children with myopia treated with topical atropine. The data is collected on 1545 eyes with spherical equivalent (SE) less than −10.0 diopters (D) treated with atropine for myopia control. Four machine learning models, namely multivariate adaptive regression splines (MARS), classification and regression tree (CART), random forest (RF), and eXtreme gradient boosting (XGBoost), were used. Linear regression (LR) was used for benchmarking. The 10-fold cross-validation method was used to estimate the performance of the five methods. The main outcome measure is that the 19 important factors associated with atropine use that may affect IOP are evaluated using machine learning models. Endpoint IOP at the last visit was set as the target variable. The results show that the top five significant variables, including baseline IOP, recruitment duration, age, total duration and previous cumulative dosage, were identified as most significant for evaluating the effect of atropine use for treating myopia on IOP. We can conclude that the use of machine learning methods to evaluate factors that affect IOP in children with myopia treated with topical atropine is promising. XGBoost is the best predictive model, and baseline IOP is the most accurate predictive factor for endpoint IOP among all machine learning approaches.

## 1. Introduction

Myopia is prevalent in the youth of many developed countries [1,2,3,4]. According to the Ministry of Health and Welfare of Taiwan, more than 80% of all school children develop myopia by the end of high school in Taiwan [5]. Since the increase in myopia cases is a worldwide trend, there are many effective mechanisms to control myopia progression such as atropine [4,6,7,8,9,10,11,12,13,14,15,16,17,18], orthokeratology [19,20], experimental bifocal/multifocal soft contact lenses (CLs) [21,22], commercial bifocal or multifocal soft CLs dedicated to presbyopia corrections but used for myopia control [23,24], and commercial multifocal CLs specially dedicated to myopia control [25,26]. Atropine, an anticholinergic blocking agent, can temporarily paralyze the smooth ciliary muscle causing cycloplegia. Historically, atropine was initially used to slow down myopia progression caused by excessive accommodation. The molecular/cellular mechanism of atropine in curtailing myopia progression has also been studied with regard to its complex interaction at the muscarinic (MRs) receptors on ocular tissues such as sclera, retina, retinal pigment epithelium, and choroid [6]. Atropine eye drops have been effective in combating myopia progression in Asian children [6,7,8,9,10,11,12,13,14,15,16,17,18]. However, long-term atropine application has raised concerns among some ophthalmologists [27,28,29]. Atropine can paralyze the pupillary muscle, increasing resistance and impeding the flow of the aqueous humor, which might increase intraocular pressure (IOP) [30,31,32,33]. We aimed to identify factors that predict incident IOP in patients with myopia after atropine use. A total of 19 factors associated with atropine use that may affect IOP were chosen for machine learning analyses [34,35,36,37]. Four machine learning techniques were used to identify factors that predict the patient’s IOP after atropine use. Our goal is to aid physicians in making decisions concerning atropine use in children with myopia by providing analytical data for clinical consideration.

## 2. Methods

In this study, four machine learning approaches, namely multivariate adaptive regression splines (MARS), classification and regression tree (CART), random forest (RF), and eXtreme gradient boosting (XGBoost), were used to construct predictive models for evaluating the effect of atropine use in myopia treatment on IOP. To evaluate the performance of the four machine learning methods, linear regression (LR), a classic prediction method, was used as a benchmark.

### 2.1. MARS

MARS is a nonparametric statistical method that can be used to divide the dataset into separate groups, with each group having its own regression equation [38]. MARS can select optimal transformations and interactions between variables and model relationships that are approximately additive or involve interactions using few variables. At different intervals of the independent variable space, separate linear regression slopes are used to approximate the nonlinearity of the MARS model. The first step involving the MARS method includes using a forward algorithm to select all possible basic functions and their corresponding knots. Then, to generate the best combination of existing knots, a backward algorithm is used to eliminate all basic functions in the order of the least contributions based on the generalized cross-validation criterion. Finally, the variables and their value for knots of hinge functions are generated.

### 2.2. CART

CART is a decision tree method developed by Breiman et al. [39]. The first step for modeling CART includes constructing a maximal tree by using binary splits; the tree can describe the training set in as much detail as possible. The next step involves trimming overgrown trees, which includes overfitting, and less complex trees. Finally, CART uses a cross-validation procedure to choose an optimal tree size. When constructing a maximal tree, binary splitting is initiated from the root node of the tree. The root node consists of all objects in the training set and uses the Gini index to measure the impurity of the split. Based on the Gini index, the parent node can divide into two exclusive child nodes. Child node selection depends on the best reduction of the Gini index between the parent and child nodes. The tree repeats previous steps to grow continuously until a significant decrease in impurity is achieved. When the condition is reached, the tree stops growing and becomes a terminal node.

### 2.3. RF

RF is an ensemble classification method developed by constructing several decision trees [40]. RF applies the general technique of bootstrap aggregation or bagging to select various random samples of variables as the training data set, and the meta-algorithm uses the replacement to synchronously reduce variance and avoid overfitting during random sampling [41]. The CART algorithm is used on RF modeling. RF uses the “out-of-the-bag” error to measure performance. It calculates the average of the error rate of each weak learner. Each tree is specifically explored in the RF method, and the most significant variables are used as nodes. Finally, each tree is grown to its maximum capacity

### 2.4. XGBoost

XGBoost is a tree-based learning algorithm that is a scalable, end-to-end, gradient tree boosting system [42]. Boosting refers to the ensemble learning technique of building many models sequentially, with each new model attempting to correct for the imperfections or inadequacies in the previous model. XGboost implements the generalized gradient boosting decision tree and uses a new distributed algorithm to speed up tree construction. To solve the overfitting issue, XGboost uses the regularization term, which controls the complexity of the model, and simultaneously uses both the first- and second-order derivatives to perform a second-order Taylor expansion of the loss function. Then, the loss function computed pseudo-residuals in the first and second derivatives generate learning.

### 2.5. Model Implementation

The methods were implemented in R software of version 3.6.2 (R core team, Vienna, Austria). The algorithms for the methods are based on the relevant R package. The default setting was used to construct the models. For the LR method, the “stats” R package, version 3.6.2, was used. The “earth” R package, version 5.1.2, proposed by Milborrow et al. [43], was used to construct the MARS model. For the CART model, the “rpart” R package, version 4.1-15, proposed by Therneau et al. [44] was employed. The “randomForest” R package, version 4.6-14, was used to construct the RF model [45]. The XGBoost model was constructed by implementing the “xgboost” R package, version 0.90.0.2 [46].

This study used the 10-fold cross validation method to estimate the performance of the five models. The performance metrics are important for measuring the prediction accuracy of each method. In this study, five performance metrics, namely the mean absolute percentage error (MAPE), symmetric MAPE (SMAPE), relative absolute error (RAE), root relative squared error (RRSE), and root mean squared error (RMSE), were used for evaluating the prediction error of each method. Table 1 summarizes the definitions of these performance metrics. MAPE, SMAPE, RAE, RRSE, and RMSE were used to evaluate deviations between actual and predicted values; the lower the deviation, the higher the accuracy. We used the “*MLmetrics*” R package, version 1.1.1, proposed by Yan [47] to generate the MAPE, RAE, RRSE, and RMSE metrics for each method. The “Metrics” R package, version 0.1.4, proposed by Hamner et al. [48] was used to compute the SMAPE value.

## 3. Empirical Study

### 3.1. Dataset and Performance Criteria

**Figure 1:**
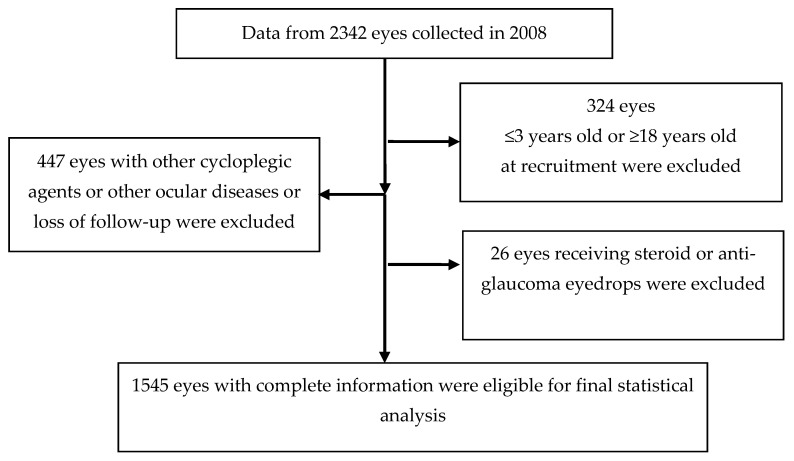
Algorithm of case identification.

Data from 2342 eyes of 1171 children with myopia and a refractive error of spherical equivalent (SE) less than −10.0 D diagnosed at the Shin-Kong Wu Ho-Su Memorial Hospital (SK Hospital) in Taipei, Taiwan, between 1 January 2008, and 31 December 2008 were included in this study. We excluded 324 eyes from participants younger than three years or older than 18 years. A total of 447 eyes were further excluded due to loss of follow-up; use of cycloplegics other than atropine; ocular diseases including corneal opacity, traumatic injury, uveitis, congenital cataract, congenital glaucoma, optic nerve atrophy, and ocular tumor; or surgery. Moreover, 26 eyes were excluded due to the use of steroids or antiglaucoma medications. Finally, data from 1545 eyes were used for analyses (Figure 1). With respect to ethical issues regarding usage of dataset, the protocol of this study was evaluated and deemed acceptable by the Research Ethics Review Committee at the Shin Kong Wu Ho-Su Memorial Hospital (IRB No. 20200205R).

Of the 1545 eyes, 813 and 732 from male and female children, respectively, we reviewed medical records, refractive status, and the duration and dosage of atropine treatment. Table 2 lists 19 factors (variables X1–X19) associated with atropine use that may affect IOP. From 1 January 2008, to 31 December 2008, the measurements of initial IOP and refraction were variable X3 (base IOP) and X4–X7, respectively. IOP was measured in both eyes, beginning with the right eye. Noncontact tonometry (Xpert NCT plus, Reichert, Leica Inc.) was used without topical anesthesia in the seated position. The refractory error was obtained using a Canon RK5 autorefractor auto-keratometer (Canon Inc. Ltd., Tochigiken, Japan).

Our study is a retrospective study. The data we reviewed are from the first medical treatment day after 1 January 2005 as the first visit, and the patient record was traced to 30 December 2008 as the last clinic visit. This period is the total duration (X8). Prescribed atropine dosages were calculated by multiplying the dosage on the prescription bottles (50, 25, 12.5, or 5 mg) with the number of bottles prescribed (X11). A cumulative dosage is the sum of all prescribed dosages during outpatient visits for a patient in the given time.

Use of atropine from 1 January 2005 to 31 December 2007 was incorporated as “previous data” (X12–X14), which included previous durations, previous cumulative dosages, and previous average dosages per month. Statistics from 1 January 2008 to 31 December 2008 were incorporated as “recruit data” (X15–17), which included recruitment durations, recruit cumulative dosages, and recruit average dosages per month. Furthermore, “total duration” (X8) included the sum of data for the previous duration and recruitment duration. Thus, the previous cumulative dosage and recruit cumulative dosage constitute the ‘‘total cumulative dosage” (X9). The prescribed doses of atropine from the last visit of the patients before the termination of data collection were included as the “last dosage” (X18); the prescribed frequency of atropine from the last visit of the patients before the termination of data collection was included as the “last frequency” (X19), which was recorded as QN (every night), QON (every other night), BIW (twice a week), QW (once a week), and 0 (none prescribed). The endpoint IOP of the last visit before the termination of data collection was set as the variable Y.

### 3.2. Results

The 19 variables (Xs) considered as impact factors for the development of IOP (Y) in children with myopia due to atropine use are shown in Table 3. The gender (X1) distribution of the 1545 eyes was 52.6% and 47.4% for 813 and 732 male and female children, respectively. The baseline age (X2) of our participants was 10.53 ± 2.54 years. The total duration (X8) of atropine intervention, defined as the time lapsed from the first visit after 1 January 2005, to the last visit before 31 December 2008, was 20.02 ± 12.01 months. Baseline IOP (X3) was 14.51 ± 2.69 mmHg at the first visit. The value of endpoint IOP measured at the end of the total duration (Y) was 15.08 ± 2.86 mmHg.

Baseline myopic power (X4) was −1.95 ± 1.45 D with a base SE (X5) of −2.48 ± 1.57 D. At the end of the total duration, the endpoint myopic power (X6) was −2.39 ± 1.68 D with an endpoint SE (X7) of −2.94 ± 1.86 D. The number of prescription bottles (X11) of atropine was 6.47 ± 5.54. The total cumulative dosage (X9) of atropine was 75.00 (interquartile range [IQR], 37.5–150) mg. The total average dosage of atropine per month (X10) was 4.35 (IQR, 2.66–7.87) mg.

The previous duration (X12), defined as the time interval from the first visit after 1 January 2005 to the last visit before 31 December 2007, was 14.07 ± 12.14 months. The previous cumulative dosage of atropine (X13) was 45.00 (IQR, 25–87.5) mg. The previous average dosage of atropine per month (X14) was 6.42 (IQR, 2.34–15.99) mg.

The duration between the first visit after 1 January 2008 and the last visit before 31 December 2008, defined as the recruitment duration (X15), was 5.96 ± 3.83 months. The recruitment cumulative dosage of atropine (X16) was 12.50 (IQR, 0–55) mg. The recruit average dosage of atropine per month (X17) was 2.35 (IQR, 0–11.93) mg.

The dosages of atropine prescribed at the last visit (X18) before 31 December 2008 were as follows: 4 (0.3%) eyes were not prescribed atropine; 619 (40.1%) eyes, 5 mg (0.1%) atropine; 718 (46.5%) eyes, 12.5 mg (0.25%); 146 (9.4%) eyes, 25 mg (0.5%); and 58 (3.8%) eyes, 50 mg (1%).

The last frequency (X19) describes instructions for atropine application at the last visit before the termination of data collection. A total of 1191 (77.1%) eyes were instructed to apply atropine every night (QN); 238 (15.4%) eyes, every other night (QON); 80 (5.2%) eyes, twice a week (BIW); and 32 (2.1%) eyes, once a week (QW).

We used LR, MARS, CART, RF, and XGBoost to construct predictive models. The MAPE, SMAPE, RAE, RRSE and RMSE values of XGBoost were found to be 0.1182, 0.1155, 0.7783, 0.8211, and 2.2604, respectively (Table 4). The XGBoost method generated the lowest value of all metrics compared with the LR, MARS, CART, and RF methods. Thus, XGBoost was the best predictive model in this study and outperformed other methods.

In addition to evaluating the performance of each method, ranking the importance of each variable within different models can provide useful information for identifying important risk factors. To rank the relative importance of each variable, the “caret” R package of version 6.0-84 [49] based on the Wrapper method was used for each of the five methods. The most important predictor was ranked 1. By contrast, the predictor with the lowest importance was ranked the last.

**Figure 2:**
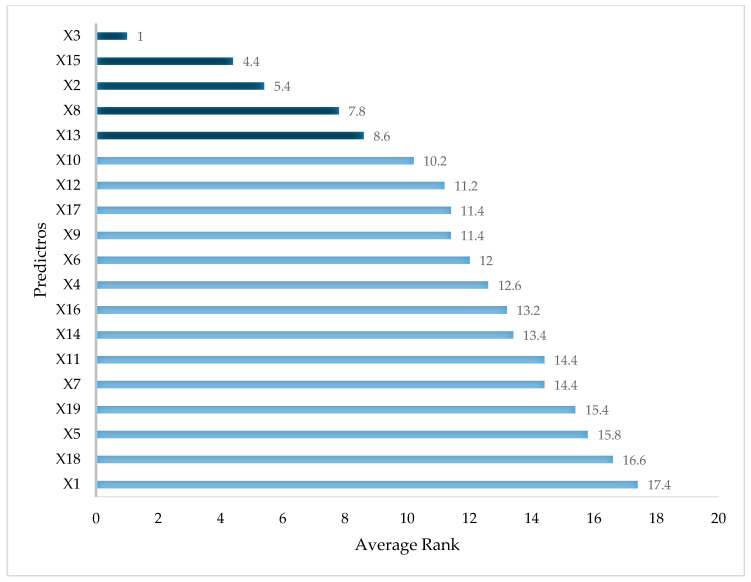
Ranking of all predictors.

Table 5 shows the importance rank of each predictor by using the LR, MARS, CART, RF, and XGBoost methods, respectively. Baseline IOP (X3) was found to be the most important variable in the LR model, followed by the total cumulative dosage (X9) and previous cumulative dosage (X13). However, different methods generated different relative importance ranks of each variable. In the XGBoost model, the most important variable was baseline IOP (X3). However, the second- and third-most important variables were age (X2) and the previous average dosage per month (X14), respectively. For the overall importance of variables, we averaged the rank value of each variable in each method. Figure 2 depicts the ranked overall variable importance of all predictors.

To simplify the discussion of predictor importance, more important predictors were selected based on the average rank of each variable, as shown in Figure 2. According to suggestions from physicians, the top five important variables, namely baseline IOP (X3), recruitment duration (X15), age (X2), total duration (X8), and previous cumulative dosage (X13), were selected as significant variables for evaluating their effect on IOP in myopia control with topical atropine.

## 4. Discussion

The effectiveness of atropine in myopia treatment has been extensively discussed [6,7,8,9,10,11,12,13,14,15,16]. Studies have evaluated the ability of atropine in myopia control and its side effects at different concentrations [17,18]. Atropine, an anticholinergic that causes pupillary dilation, is contraindicated in glaucoma [31,32,33,34]. Because of a shallow anterior chamber in children, ophthalmologists have a concern regarding whether using atropine for treating children with myopia will affect their IOP due to the induction of mydriasis by muscarinic antagonists [27,28,29]. We used clinical data combined with machine learning analyses to predict 19 factors that may affect IOP in children with myopia treated with atropine. Baseline IOP (X3), recruitment duration (X15), age (X2), total duration (X8), and previous cumulative dosage (X13) were the top five significant variables for evaluating the effect on the IOP of children with myopia treated with topical atropine.

Baseline IOP (X3) is the most accurate predictive factor for endpoint IOP among all machine learning approaches. IOP in children increased with age (X2) and reached levels similar to those in adults by the age of 12 years [50]. Myopia control with atropine for children may start as children enter school at around six years of age and extend up to the age of 16 years [16,17,18,27]. Ophthalmologists should consider an increase in IOP with age when prescribing atropine to school-aged children.

Baseline IOP should be measured prior to atropine prescription, and the measurement should be compared with the normal value for children of the same age as the patient [37,50]. If baseline IOP is considerably higher, the patient is at a higher risk of high endpoint IOP later than patients with normal IOP. We suggest that atropine prescription should not be based on myopic power alone; clinicians should also take IOP into account. Meanwhile, basal IOP is related to the corneal pachymetry and corneal thickness should be used as a parameter for pachymetry-corrected basal IOP [51,52]. Most of our subjects in this study are children age 3 to 18 years old, but some of them cannot cooperate with the measurement of corneal pachymetry. In future studies, if we can collect more data from cooperative patients, the correlation between IOP and corneal thickness should also be considered. The follow-up period between the baseline and final IOP was about two years. As the children grew older, their coordination degree would be better, so this might affect the measurement values of basal and final IOP. This time, we measured the IOP only with a non-contact pneumatic tonometer. However, different instruments may also present the intrasession and intersession fluctuations of IOP. Therefore, we should consider integrating or comparing the fluctuations of baseline and final IOP in the future [53].

The recruitment duration (X15) and total duration (X8) are significant factors predicting endpoint IOP. The recruitment duration (X15), one year before the termination of data collection, reflects the frequency and number of visits, which is indicative of a patient’s approach to medical care [30,31,32]. Because only for eyes were not prescribed atropine during recruitment, data from the recruitment duration can be considered as being from patients that had been taking atropine during the previous year. The more atropine usage within one year, the more attention needs to be given to the effect of atropine on the patient’s IOP. Furthermore, the total duration (X8) is related to high IOP in children with myopia because total time-lapse exhibited the onset of myopia and atropine exposure. A longer total duration suggests that the patient had myopia onset at a younger age and was using atropine for an extended period [32,33,34,35]. The total duration of atropine usage should be considered when following IOP in children with myopia.

The previous cumulative dosage (X13) is the sum of all prescribed dosages to children with myopia who use atropine. Higher previous cumulative dosages may represent rapidly progressive myopia and stronger atropine dose control, or an earlier onset of myopia and atropine use for a long time. Some of the variables such as dosage depend on the patient’s strict compliance with the administration of the drug. Therefore, children with myopia treated with atropine should be evaluated for IOP.

Based on previous clinical studies, the author has published the clinical literature on the use of atropine on myopic children in this group. The clinical discussion revealed the relevant factors such as age, dosage, use time, etc., and whether the changes of IOP will be caused by atropine before and after its use [29]. At present, other literature has discussed the clinical effects or side effects of different doses of atropine [37,54]. The new significance of this study of atropine in myopic children is the use of the clinical data, combined with four artificial intelligence methods, to find out the reliable predictive value and the important rank of factors which may be related to topical atropine use for myopia control on intraocular pressure. In this study, machine learning was used to analyse 19 variables associated with the effect of atropine on IOP. However, these variables were chosen based on clinical data and the pharmacological properties of atropine, and other variables such as dose and period of treating can be analysed in the future. In addition, analysis of the top five significant variables revealed predictor importance and not the positive or negative correlation parameters or absolute data for these variables. Future studies should identify the “Baseline IOP,” which was the most important predictive variable in our study and identify a safe range for atropine use in children with myopia in the clinical setting.

## 5. Conclusions

The 19 factors that might estimate the risk of high IOP in children with myopia who use atropine were evaluated using machine learning methods. We implemented four machine learning methods to quantify each factor and its relative association with the risk of high IOP. Baseline IOP, recruitment duration, age, total duration, and previous cumulative dosage were the top five significant variables for endpoint IOP. Our findings from machine learning provide clinicians with information to evaluate the suitability of a patient for atropine treatment. We wish to highlight different factors that may contribute to IOP in patients receiving atropine treatment.

## Figures and Tables

**Table 1:** Equation of performance metrics.

Metrics	Description	Calculation *
MAPE	Mean Absolute Percentage Error	$MAPE=\frac{1}{m}\overset{m}{\underset{j=1}{\sum }}\left|\frac{p_{j}-q_{j}}{q_{i}}\right|\times 100$
SMAPE	Symmetric Mean Absolute Percentage Error	$SMAPE=\frac{1}{m}\overset{m}{\underset{j=1}{\sum }}\frac{\left|p_{j}-q_{j}\right|}{\left(\left|p_{j}\right|+\left|q_{j}\right|\right)/2}$
RAE	Relative Absolute Error	$RAE=\frac{\sum_{j=1}^{m}\left|p_{j}-q_{j}\right|}{\sum_{j=1}^{m}\left|q_{j}-\bar{q}\right|}$
RRSE	Root Relative Squared Error	$RRSE=\sqrt{\frac{\sum_{j=1}^{m}{\left(p_{j}-q_{j}\right)}^{2}}{\sum_{j=1}^{m}{\left(q_{j}-\bar{q}\right)}^{2}}}$
RMSE	Root Mean Squared Error	$RMSE=\sqrt{\frac{1}{m}\overset{m}{\underset{j=1}{\sum }}{\left(p_{j}-q_{j}\right)}^{2}}$

* Note: *p* and *q* represent predicted and actual values, respectively; *m* is the total amount of data.

**Table 2:** Variable definition.

Variables		Description	Unit
X1:	Sex	Male/Female	-
X2:	Age	Age in years	-
X3:	Base IOP	Baseline intraocular pressure measured	mm-Hg
X4:	Base Spherical	Baseline myopic power	Diopter (D)
X5:	Base SE	Baseline spherical equivalent	Diopter (D)
X6	End Spherical	Endpoint myopic power	Diopter (D)
X7	End SE	Endpoint spherical equivalent	Diopter (D)
X8	Total Duration	Total duration from first to last visit	month
X9	Total Cumulative Dosage	Total cumulative dosage of topical atropine	mg
X10	Total Average Dosage per month	Average dosage per month from the first visit to the last visit	mg/month
X11	Total Prescribed Bottles	Number of prescription bottles of atropine	bottles
X12	Previous Duration	The duration from the first visit to the recruitment date	month
X13	Previous Cumulative Dosage	Cumulative dosage of topical atropine from the first visit to the recruitment date	mg
X14	Previous Average Dosage Per Month	Average dosage per month from the first visit to the recruitment date	mg/month
X15	Recruit Duration	The duration from the recruitment date to the last visit	month
X16	Recruit Cumulative Dosage	Cumulative dosage of topical atropine from the recruitment date to the last visit	mg
X17	Recruit Average Dosage Per Month	Average dosage per month from the recruitment date to the last visit	mg/month
X18	Last Dosage	The prescribed dosage of atropine on the last visit	mg
X19	Last Frequency	The prescribed frequency of atropine on the last visit	-
Y	End IOP	Endpoint intraocular pressure measured	mm-Hg

Note: IOP: intraocular pressure; SE: spherical equivalent.

**Table 3:** Participant demographics.

Characteristics		Mean ± SD
X2	Age	10.53 ± 2.54
X3	Base IOP (mm-Hg)	14.51 ± 2.69
X4	Base Spherical (Diopter (D)	−1.95 ± 1.45
X5	Base SE (Diopter (D)	−2.48 ± 1.57
X6	End Spherical (Diopter (D)	−2.39 ± 1.68
X7	End SE (Diopter (D)	−2.94 ± 1.86
X8	Total Duration (month)	20.02 ± 12.01
X11	Total Prescribed Bottles	6.47 ± 5.54
X12	Previous Duration (mg)	14.07 ± 12.14
X15	Recruit Duration (month)	5.96 ± 3.83
Y	End IOP (mm-Hg)	15.08 ± 2.86
		**Median (IQR)**
X9	Total Cumulative Dosage (mg)	75.00 (37.5–150)
X10	Total Average Dosage per Month (mg/month)	4.35 (2.66–7.87)
X13	Previous Cumulative Dosage (mg)	45.00 (25–87.5)
X14	Previous Average Dosage Per Month (mg/month)	6.42 (2.34–15.99)
X16	Recruit Cumulative Dosage (mg)	12.50 (0–55)
X17	Recruit Average Dosage Per Month (mg/month)	2.35 (0–11.93)
X1	Sex	***n* (%)**
	Male	813 (52.6%)
	Female	732 (47.4%)
X18	Last Dosage (mg)	***n* (%)**
	0	4 (0.3%)
	5	619 (40.1%)
	12.5	718 (46.5%)
	25	146 (9.4%)
	50	58 (3.8%)
X19	Last Frequency	***n* (%)**
	QN	1191 (77.1%)
	QON	238 (15.4%)
	BIW	80 (5.2%)
	QW	32 (2.1%)
	0	4 (0.3%)

Note: SD: standard deviation; IQR: interquartile range; IOP: intraocular pressure; SE: spherical equivalent; QN: every night; QON: every other night; BIW: twice a week; QW: once a week.

**Table 4:** Model performance of LR, MARS, CART, RF, and XGBoost methods.

Methods	MAPE	SMAPE	RAE	RRSE	RMSE
LR	0.1266	0.1226	0.8235	0.8458	2.3283
MARS	0.1259	0.1219	0.8228	0.8409	2.3149
CART	0.1341	0.1298	0.8666	0.8835	2.4323
RF	0.1271	0.1229	0.8222	0.8331	2.2934
XGBoost	0.1182	0.1155	0.7783	0.8211	2.2604

Note: LR: linear regression; MARS: multivariate adaptive regression splines; CART: classification and regression tree; RF: random forest; XGBoost: eXtreme gradient boosting; MAPE: mean absolute percentage error; SMAPE: symmetric mean absolute percentage error; RAE: relative absolute error; RRSE: root relative squared error; RMSE: root mean squared error.

**Table 5:** Importance rank of each predictor using the five methods.

Predictors	LR	MARS	CART	RF	XGBoost	Average Rank
X1 Sex	11	19	19	19	19	17.4
X2 Age	16	4	2	3	2	5.4
X3 Base IOP	1	1	1	1	1	1
X4 Base Spherical	12	5	19	14	13	12.6
X5 Base SE	14	19	19	13	14	15.8
X6 End Spherical	8	5	19	11	17	12
X7 End SE	13	19	19	12	9	14.4
X8 Total Duration	5	19	5	4	6	7.8
X9 Total Cumulative Dosage	2	19	19	6	11	11.4
X10 Total Average Dosage per month	15	19	4	8	5	10.2
X11 Total Prescribed Bottles	9	19	19	10	15	14.4
X12 Previous Duration	5	19	19	5	8	11.2
X13 Previous Cumulative Dosage	3	19	7	2	12	8.6
X14 Previous Average Dosage Per Month	19	19	19	7	3	13.4
X15 Recruit Duration	4	2	3	9	4	4.4
X16 Recruit Cumulative Dosage	19	3	19	15	10	13.2
X17 Recruit Average Dosage Per Month	7	19	6	18	7	11.4
X18 Last Dosage	10	19	19	17	18	16.6
X19 Last Frequency	19	7	19	16	16	15.4

Note: LR: linear regression; MARS: multivariate adaptive regression splines; CART: classification and regression tree; RF: random forest; XGBoost: eXtreme gradient boosting; IOP: intraocular pressure; SE: spherical equivalent.

## Data Availability

Data available on request due to privacy/ethical restrictions.
